# Working Condition Recognition Based on Transfer Learning and Attention Mechanism for a Rotary Kiln

**DOI:** 10.3390/e24091186

**Published:** 2022-08-25

**Authors:** Yuchao Hu, Weihua Zheng, Xin Wang, Bin Qin

**Affiliations:** 1 School of Electrical & Information Engineering, Hunan University of Technology, Zhuzhou 412007, China; 2 College of Railway Transportation, Hunan University of Technology, Zhuzhou 412007, China

**Keywords:** rotary kiln, flame image, working condition recognition, deep learning, transfer learning, coordinate attention mechanism

## Abstract

It is difficult to identify the working conditions of the rotary kilns due to the harsh environment in the kilns. The flame images of the firing zone in the kilns contain a lot of working condition information, but the flame image data sample size is too small to be used to fully extract the key features. In order to solve this problem, a method combining transfer learning and attention mechanism is proposed to extract key features of flame images, in which the deep residual network is used as the backbone network, the coordinate attention module is introduced to capture the position information and channel information on the branch of feature graphs, and the features of flame images obtained are further screened to improve the extraction ability. At the same time, migration learning is performed by the pre-trained ImageNet data set, and feature migration and parameter sharing are realized to cope with the training difficulty of a small sample data size. Moreover, an activation function Mish is introduced to reduce the loss of effective information. The experimental results show that, compared with traditional methods, the working condition recognition accuracy of rotary kilns is improved by about 5% with the proposed method.

## 1. Introduction

Rotary kilns are widely used in cement, metallurgy, chemical and environmental protection fields. The firing zone temperature in the kilns is adjusted by controlling the amount of coal feeding, the damper opening and the kiln rotational speed according to the current kiln conditions during the production process. They are the most important factors in determining the clinker quality and are usually used as the controlled variables in the rotary kiln control strategies [1,2]. However, due to the influence of interfering factors, such as smoke and dust in the kilns, the traditional method is difficult to measure the temperature of the sintering zone accurately.

There is a close relationship between flame image and temperature, and the flame image of the firing zone contains a wealth of information about the operating conditions. In order to achieve intelligent control of rotary kilns, many intelligent methods, such as singular value decomposition (SVD) [3], support vector machine (SVM) and K-means [4] are applied to the feature extraction and classification of such flame images to recognize working conditions of rotary kilns.

However, machine learning and neural network approaches alone are not satisfactory in terms of recognition accuracy. As a result, most of the subsequent studies chose to pre-process the flame images by combining filters or image segmentation to highlight the regions of interest before using neural networks for working condition recognition, which improved recognition accuracy to a certain extent [5,6]. However, due to the limitation of the number of network layers and performance, these methods using neural networks are difficult to distinguish important features when extracting features from flame images with high similarity, and it is also difficult to select classifiers.

In recent years, the application of deep learning in the field of image recognition has made breakthrough progress [7]. However, it is difficult to obtain images of rotary kiln flames: the lack of sample data makes it difficult to meet the demand for image feature extraction in deep learning networks, and the training is prone to overfitting. To reduce the dependence of deep learning models on the number of training samples, transfer learning can be applied to classification recognition tasks for speeding up training efficiency [8]. Therefore, a combination of deep learning and transfer learning is applied to the recognition of rotary kiln working conditions. In [9], a convolutional neural network was used for feature migration, and the network was trained and tested for automatic recognition of combustion states. There are also methods based on deep transfer learning to obtain the feature space of the dynamic convolutional neural network and input the feature vector into stochastic configuration networks (SCN) to realize the recognition of the burning state of the flame image [10]. These methods have alleviated the problem of a lack of sample data for flame images to some extent, but due to the high similarity between different classes of flame images of rotary kilns, the existing deep learning models do not pay special attention to the important image features, resulting in difficulties in extracting key features during the training process.

In order to improve the ability of deep learning networks to extract key features, an attention mechanism [11,12] is added to the computer vision research to complement and improve existing deep learning networks, which is popular in natural language processing. The attention mechanism was first used on the RNN model, and a visual processing framework based on it was proposed [13]. In [14], in order to find the most relevant regions in the input images, a deep learning network Deep RNN and attention mechanism were combined to recognize images and reduce the image classification errors.

The method of combining attention mechanism and deep learning network has begun to be widely used in the field of computer vision [15], such as the classification and recognition of medical images [16], the classification of remote sensing images [17], and the detection of industrial casting defects [18]. Inspired by the above research, a rotary kiln condition recognition method based on transfer learning and attention mechanism is proposed. The main contributions of this work are as follows:

- Transfer learning is used to solve the problem of data acquisition difficulty of rotary kiln flame images and avoid overfitting during model training.
- A deep residual network is used to directly perform feature extraction, classification and recognition on the flame images without segmenting them.
- An attention mechanism is added during feature extraction to improve the weight of the key flame image features, solving the problem of insufficiently extracting key features with traditional methods.
- It is the first time a combination of transfer learning and attention mechanism is used to identify rotary kiln working conditions from flame images, which accelerates the model convergence speed, and improves the model recognition accuracy and generalization ability.

The other parts of the paper are organized as follows: related work is given in Section 2. Section 3 presents a method for recognition working conditions of rotary kilns based on transfer learning and attention mechanism. The results of the experiments are reported in Section 4 and discussed in Section 5. Finally, the conclusions are given in Section 6.

## 2. Related Work

Recognition of a sintering state by flame image is helpful to determine a sintering state. In [3], the authors proposed a method of rotary-kiln combustion-state identification based on singular value decomposition (SVD) and support vector machine (SVM). The flame image features between the two libraries were extracted and used as input to train the SVM classifier offline to realize the recognition of rotary kiln. In [5], the author gradually optimized the feature space of the flame image by evaluating the uncertain cognitive results of different cognitive demand values and realized the simulated feedback cognitive mode from global to local. In this study, the authors combined the coupling operation of training layer and cognitive decision layer and established a simulation feedback mechanism based on bag of words model, latent semantic analysis method and entropy theory. The average recognition accuracy of the proposed rotary-kiln combustion-state cognition method reached 92.32%. In [4], the author proposed a combustion state recognition method for cement rotary kiln based on Otsu–K-means flame image segmentation and SVM. The Otsu–K-means image segmentation method is used to achieve the effective segmentation of the flame image target region. On this basis, 10 feature parameters of the target region are extracted as the state recognition features. Then, the one minute statistical average of the extracted feature parameters was used as the input type to classify and identify the flame image, and 28 groups of samples could be correctly identified. In [19], the authors proposed an effective quality sensing feature of texture intensity (TI) to model texture, structure and naturalness of images. Using the video quality score statistics calculated by TI-NIQE as input features, the automatic visual recognition model of rotary kiln status recognition was trained. It has high prediction accuracy for rotary kiln state recognition on the benchmark data set. However, it is challenging to extract the key features of flame image efficiently.

There are also methods of feature extraction using flame video directly. In [1], the author proposed an effective model based on the spatiotemporal feature extraction of flame video. The dynamic texture descriptor 3DBLBP is used to extract the dynamic texture and flame motion from three adjacent frames of a video block. Then, the dynamic structure descriptor HOPC-TOP is used to extract the three-dimensional phase consistency information from three orthogonal planes to capture the structure and motion characteristics of the flame. Combining these two descriptors, the spatiotemporal characteristics of flame fragments are extracted to identify the burning state. In [20], the author designed a set of trajectory evolution characteristics and morphological distribution characteristics of chaotic attractors. After the chaotic attractors were reconstructed from the intensity sequence of the flame video through phase-space reconstruction, quantitative features were extracted from the recursive graph and morphological distribution, and then put into the decision tree to identify the burning conditions. The authors claim that the method is more than 5% more accurate than other methods.

Because the flame image is difficult to obtain, it is sometimes necessary to solve the problem of uneven data sets. The authors in [2] proposed a comprehensive framework considering class disequilibrium for sintering condition recognition. The characteristics of heat signal were analyzed by the Lipschitz method, and four discriminative features were extracted to comprehensively describe different sintering conditions. A new recognition model, kernel modified ODM (KMODM), was proposed for the identification of a sintering state, which alleviated the decrease in the accuracy of minority detection. For a sintering state identification under class imbalance, a cascade stack autoencoder (SAE) model [21] was proposed to integrate prior knowledge and hidden information, extract hidden information from thermal signals, and extract discriminant features of unbalanced data. At the same time, the kernel modified Optimal Margin distribution machine (ddKMODM) is used as the sintering state recognition model, and the overall sintering state recognition accuracy of the scheme is more than 92%.

In order to meet the requirements of deep learning on the number of training samples, a rotary kiln recognition method combining transfer learning has emerged. In [10], the author proposed an intelligent sensing model of flame image combustion condition based on deep transfer learning. On the one hand, the adaptive structure convolutional neural networks (ASCNN) were constructed by a self-optimizing adjustment mechanism. On the other hand, stochastic configuration networks (SCN) with stochastic approximation ability are constructed. Experimental results demonstrate the feasibility of the recognition model. In [9], the author adopted the convolutional neural network VGG-16 model for feature transfer, and trained and tested the flame images of different combustion states in the rotary kiln through the network so as to achieve the purpose of automatic identification of combustion states. However, the application of deep learning in rotary kiln working condition recognition still has great development potential.

## 3. Rotary Kiln Working Condition Recognition Based on Transfer Learning and Attention Mechanism

The working conditions in rotary kilns are generally divided into three types: under-burning state, normal-burning state and over-burning state. The flame brightness is low, and the shiny area is small when it is under the “under-burning state”; the flame area has uniform brightness when it is under the “normal-burning state”; the flame has a high brightness and a large shiny area, and even exposure occurs, when it is under the “over-burning state”. In order to extract useful information features and improve the recognition accuracy of the working conditions, a method for identifying the working conditions of the rotary kiln based on transfer learning and attention mechanism is proposed in the paper.

**Figure 1:**
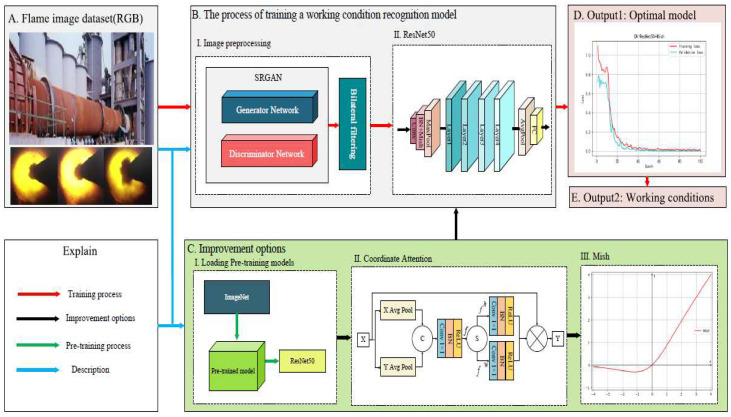
Workflow and methodology of rotary kiln working condition recognition model.

Figure 1 shows the workflow and methodology of our proposed work. It includes five parts: A: Rotary kiln flame image dataset is prepared for inputs. B: The flame images are preprocessed using bilateral filtering [22] and their resolution are improved through super resolution generative adversarial network (SRGAN) [23]. C: The pre-trained model of ResNet50 [24] is loaded for feature migration, and the coordinate attention module is embedded in the fully connected layer of the network model to improve the weight of key features in the network; its activation function Mish [25] is optimized to reduce the loss of effective information. D: The optimal model is saved. E: The corresponding rotary kiln working condition category is outputted.

### 3.1. Image Preprocessing

**Figure 2:**
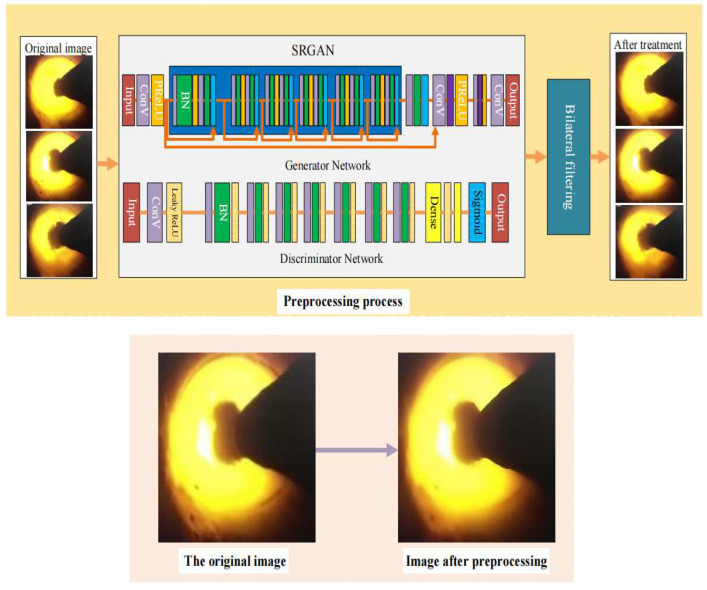
Image preprocess and its effect.

The original rotary kiln flame image data is collected by the kiln head camera. In order to make the image dataset better adapt to the recognition model, the flame images need to be preprocessed. The comparison before and after image preprocessing is shown in Figure 2. Firstly, the resolution of the flame images is improved to 224 × 224 by super resolution generative adversarial network (SRGAN). The bilateral filtering is then used to refine the edge features of flame images due to the large similarity of flame features under the three working conditions.

SRGAN consists of a generator network and a discriminant network. The real images, fake images and their corresponding labels are inputs to the discriminant network. The fake images are generated by sending low-resolution images into the generator network, and the authenticity of the input images is judged by the discriminant network through training. The images are further trained by the generator network according to the discriminator’s results. At the same time, the real high-resolution images and the fake high-resolution images are sent to the VGG network by the generator to obtain the features of the two kinds of images, and the loss is obtained by comparing them. The perceptual loss $l^{SR}$ is used to improve the realism of the recovered images and their resolution, and retain high-frequency details, which is beneficial to the identification of working conditions. The perceptual loss $l^{SR}$ consists of the content loss $l_{MSE}^{SR}$ and the adversarial loss $l_{Gen}^{SR}$.

(1) $$l^{SR}=l_{MSE}^{SR}+10^{-3}l_{Gen}^{SR}$$

(2) $$l_{MSE}^{SR}=\frac{1}{r^{2}WH}\overset{rW}{\underset{x=1}{\sum }}\overset{rH}{\underset{y=1}{\sum }}{\left(I_{x,y}^{HR}-G_{\theta_{G}}{\left(I^{LR}\right)}_{x,y}\right)}^{2}$$

where r is the amplification factor. W,H are the width and height of the images, respectively. $I^{HR}$ is the high-resolution image and G and $\theta_{G}$ represent the generator network and network parameters.

(3) $$l_{Gen}^{SR}=\sum^{\;}-lnD_{\theta_{D}}\left(G_{\theta_{G}}\left(I^{LR}\right)\right)$$

where $I^{LR}$ represents low resolution image, D represents discriminant network and $\theta_{D}$ is discriminant network parameter.

### 3.2. Transfer Learning

Transfer learning is an effective way to improve model performance when the number of training data is insufficient, especially when small sample data size is used in network models with complex structures and train difficulty [26].

**Figure 3:**
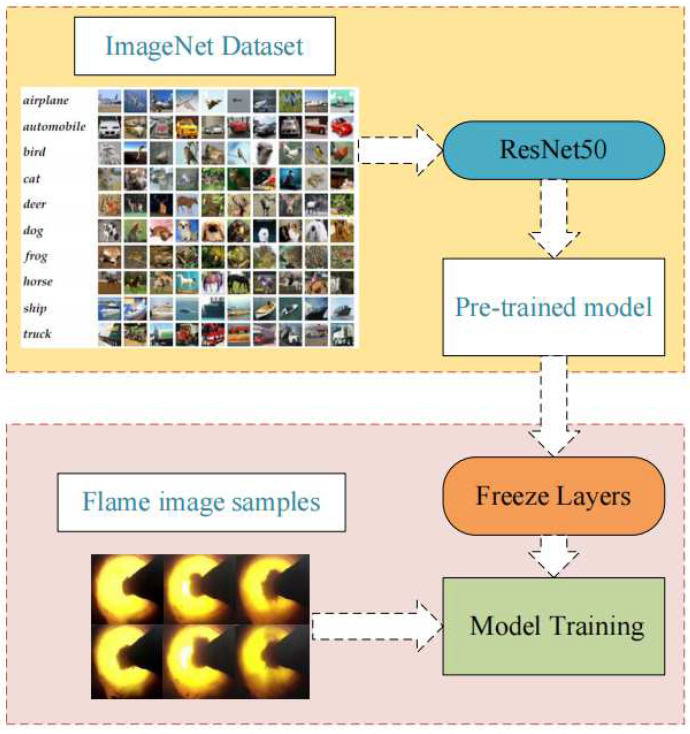
Application of transfer learning during model training.

Figure 3 shows the transfer learning approach. The basic network model is pre-trained using ImageNet dataset, and the pre-trained model is obtained by learning a large number of common underlying image features. However, as the ImageNet data set is not a special flame image data set, on the basis of the training model of the flame image, recognition is not targeted. Consequently, in the process of model training, the first step is to freeze the model of the backbone network, and then the flame image sample is used to fine-tune and train the model.

### 3.3. Coordinate Attention Module

**Figure 4:**
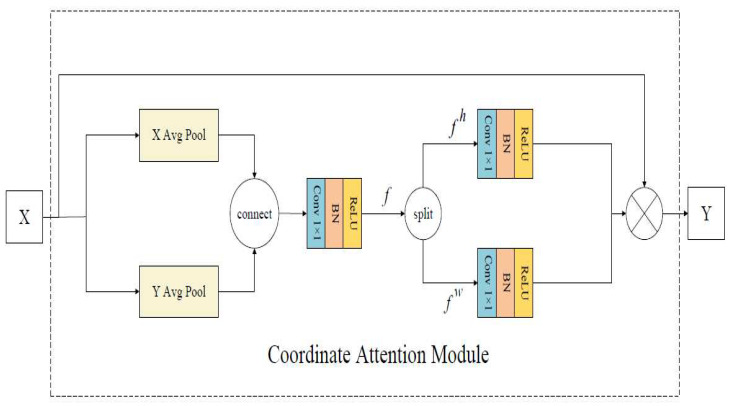
Coordinate attention module structure.

The Coordinate Attention (CA) module [27] is a lightweight attention mechanism. The location information is embedded into the channel attention to capture accurate location information and areas of interest when it acquires the feature information between channels. The specific structure of the CA module is shown in Figure 4.

The input feature map X uses the adaptive average pooling layer in the horizontal and vertical directions to extract features for each feature channel, and the generated feature maps are connected. Then, 1 × 1 convolution is used to generate the intermediate feature map with vertical and horizontal spatial information, and the intermediate feature map is divided into two feature maps along the spatial direction. Then 1 × 1 convolution is used to transform the attention weights in vertical and horizontal spatial directions, which are multiplied with the input feature map to obtain the feature map Y with the attention weight.

The CA module is introduced to improve ResNet50, and the location information is embedded in the channel attention so that the network can obtain the feature information between each channel, and improve the ability of the model to extract the key features of the flame image.

**Figure 5:**
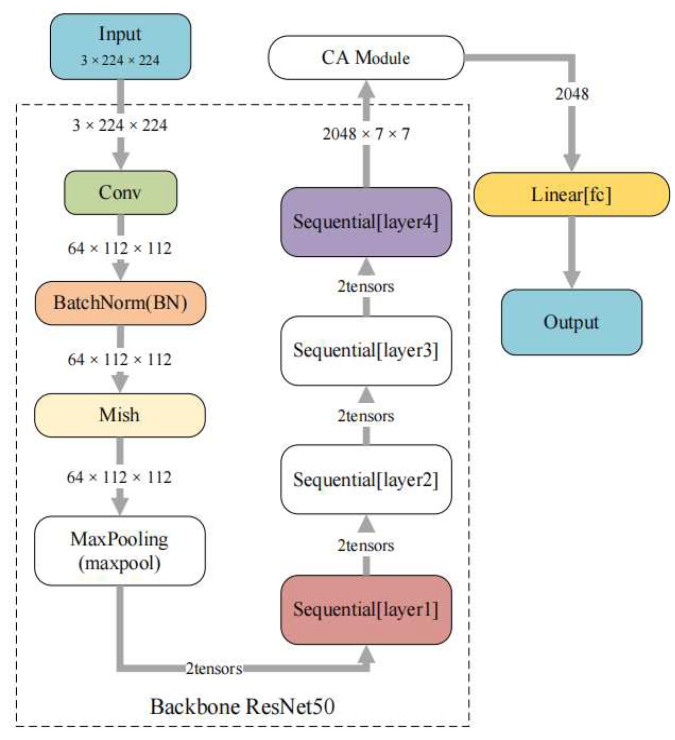
Improved ResNet50 network.

The improved ResNet50 network is shown in Figure 5. Its input is a 3 × 224 × 224 flame image, which first goes through the convolutional layers (Conv) and batch normalization (BN). Next, is the max pooling after activation with the Mish function and is then passed through the four residual modules of ResNet50 in turn. This process is simplified to Layer1 through Layer4. Then, the CA module is used for processing before the full connection layer (fc), and finally the output, is obtained.

## 4. Experiments

### 4.1. Dataset Description

The experimental data set came from the image acquisition of the on-site firing zone of an industrial rotary kiln. The number of samples in each category is balanced, and the labels are all calibrated by experts. As shown in Table 1, samples are divided into a training set and test set for model training, and we only take 20% as the test set.

### 4.2. Experimental Setup

Pytorch architecture and GPU were adopted. An AdamW optimizer and cross-entropy loss function were used. Frozen training was adopted, and the optimal model was automatically saved according to the model performance on the test set during the training process. The specific experimental parameter settings are shown in Table 2.

In the experiment, the top-1 accuracy rate is used as the evaluation index of the overall accuracy rate, and its calculation is shown as Equation (4).

(4) $$top-1\;accuracy=\frac{\mathrm{num}\left({\mathrm{test}}_{\mathrm{pred}}={\mathrm{test}}_{\mathrm{ture}}\right)}{\mathrm{num}\left(\mathrm{test}\right)}\times 100\%$$

where ${\mathrm{test}}_{\mathrm{pred}}$ represents the classification results of the test set images judged by the model; ${\mathrm{test}}_{\mathrm{ture}}$ is the label category of the test set image; $\mathrm{num}\left({\mathrm{test}}_{\mathrm{pred}}={\mathrm{test}}_{\mathrm{ture}}\right)$ is the number of correctly recognized images; and num(test) is the total number of samples in the test set. The *top*-1 *accuracy* rate is the percentage of the correct images identified in the test set, that is the recognition effect of the model on the test dataset.

To measure the performance of each model, various parameters were considered, such as precision, sensitivity, specificity and F1 score.

(5) $$Sensitivity=\frac{TP}{TP+FN}$$

(6) $$Specificity=\frac{TN}{TN+FP}$$

(7) $$Precision=\frac{TP}{TP+FP}$$

(8) $$F1\;Score=\frac{\left(2*TP\right)}{\left(2*TP+FN+FP\right)}$$

In the multi-category identification task, each category was individually considered “positive”, and all other categories were considered “negative”. For convenience of calculation, the prediction result is defined as positive, and the actual result is positive, which is represented by *TP*. If the predicted result is positive and the actual result is negative, it is represented by *FP*. If the predicted result is negative, and the actual result is positive, represented by *FN*. If the predicted result is negative, the actual result is negative, it is denoted by *TN*.

Experiments and visual analysis were carried out to verify the proposed strategy. The same rotary kiln flame image data set was used for training and testing. The pre-training model was loaded to accelerate the convergence. During the first 10 epochs, the backbone of the network was frozen for fine-tuning, and the backbone network was thawed in the subsequent epochs.

### 4.3. Experimental Results

The experiment is divided into two parts. The first part is to compare ResNet50 with different attention mechanism modules, including SE (Squeeze-and-Excitation) [28], CBAM (Convolutional Block Attention Module) [29], ECA (Efficient Channel Attention) [30] and CA module.

**Figure 6:**
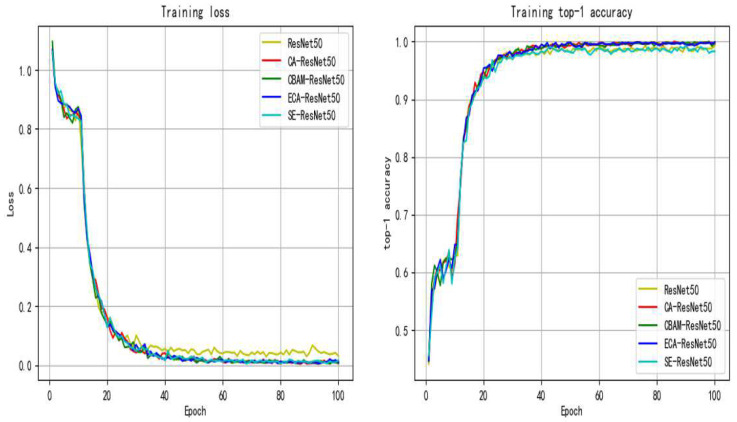
Training process curves with different attention modules.

The training process of ResNet50 after adding different attention modules is shown in Figure 6. It can be seen that the loss curves drop faster than ResNet50 after adding several different attention modules and the top-1 accuracy of ResNet50 with different attention modules except CBAM is better than that of ResNet50. The test results are shown in Table 3.

**Figure 7:**
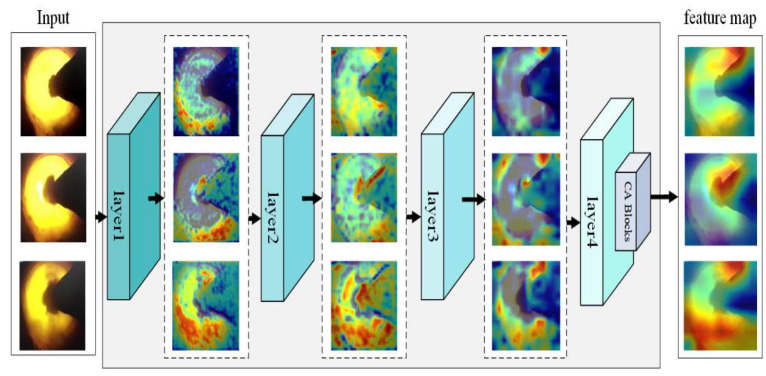
Grad-CAM visualization results of CA-ResNet50.

To further explore the effectiveness of the attention module, the visualization tool Gradient-weighted Class Activation Mapping (Grad-CAM) [31] was used to visualize the feature extraction of the network. The higher the brightness of an area on the heatmap, the higher the network pays attention to this area of the flame images. Different types of flame images were input to CA-ResNet50 respectively, and the feature extraction in several stages was obtained using Grad-CAM. The visualization results are shown in Figure 7.

**Figure 8:**
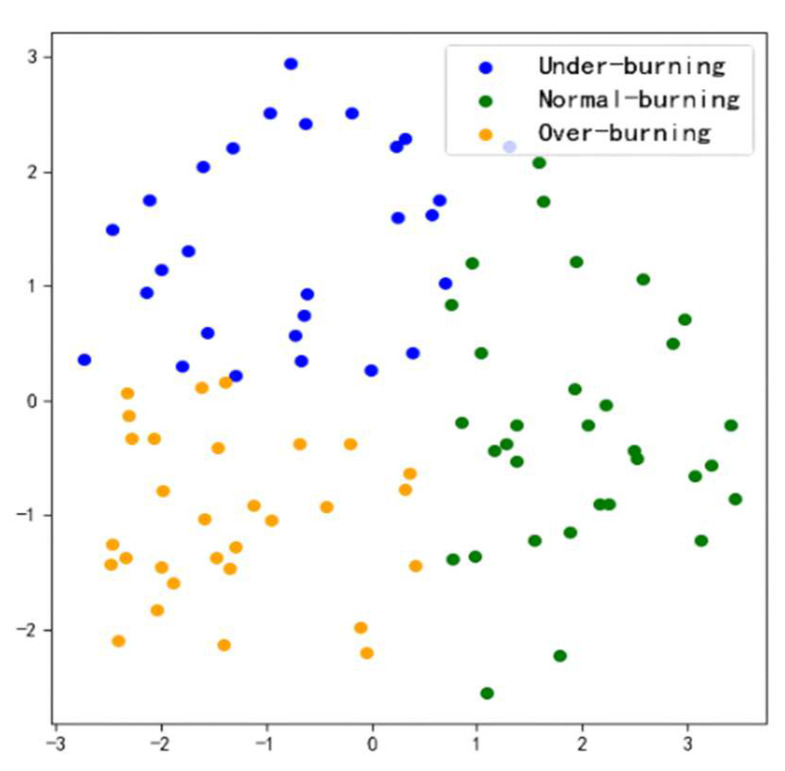
Feature space mapping of CA-ResNet50.

At the same time, a principal component analysis (PCA) is used to reduce the dimension of the test data features classified by CA-ResNet50. The two-dimensional space mapping of the test data features on CA-ResNet50 is shown in Figure 8. Data features of different categories are represented by dots of different colors in the figure. It can be seen that the classification of different categories of data features is good, indicating the feasibility of adding an attention module scheme to ResNet50.

**Figure 9:**
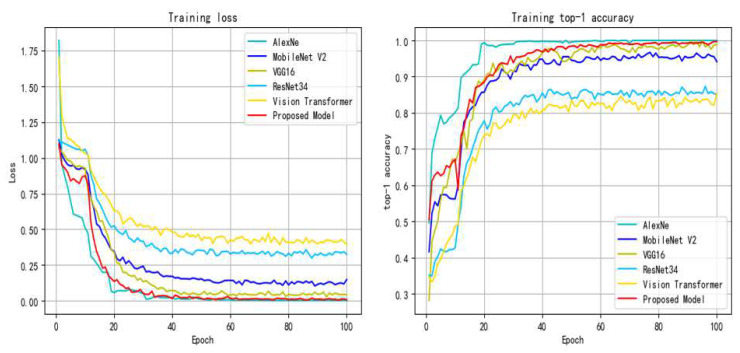
Curve comparison of different models during training process.

The second part is to compare the proposed model with different network models, including AlexNet [32], VGG16 [33], MobileNetV2 [34], ResNet34 and Vision Transformer [35]. Their training process curves are shown in Figure 9.

**Figure 10:**
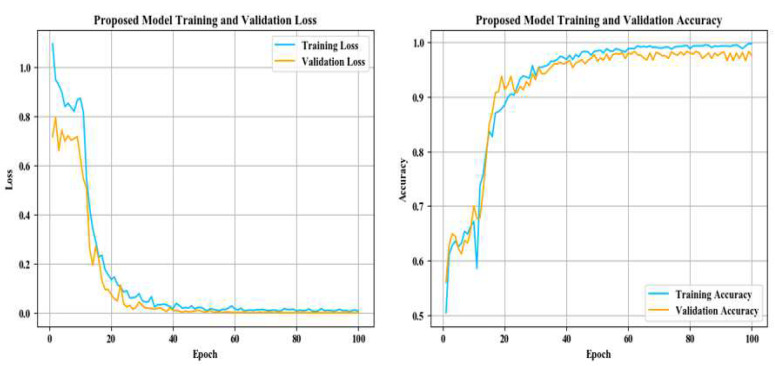
Proposed model training accuracy and loss.

Figure 10 shows the training accuracy and loss of the proposed model.

As can be seen from the changes in the loss and accuracy of the training set and validation set during model training in Figure 10, the loss of the training set and validation set shows a downward trend. Combined with the performance of the proposed model in accuracy, it is shown that the proposed model does not have the problem of overfitting.

**Figure 11:**
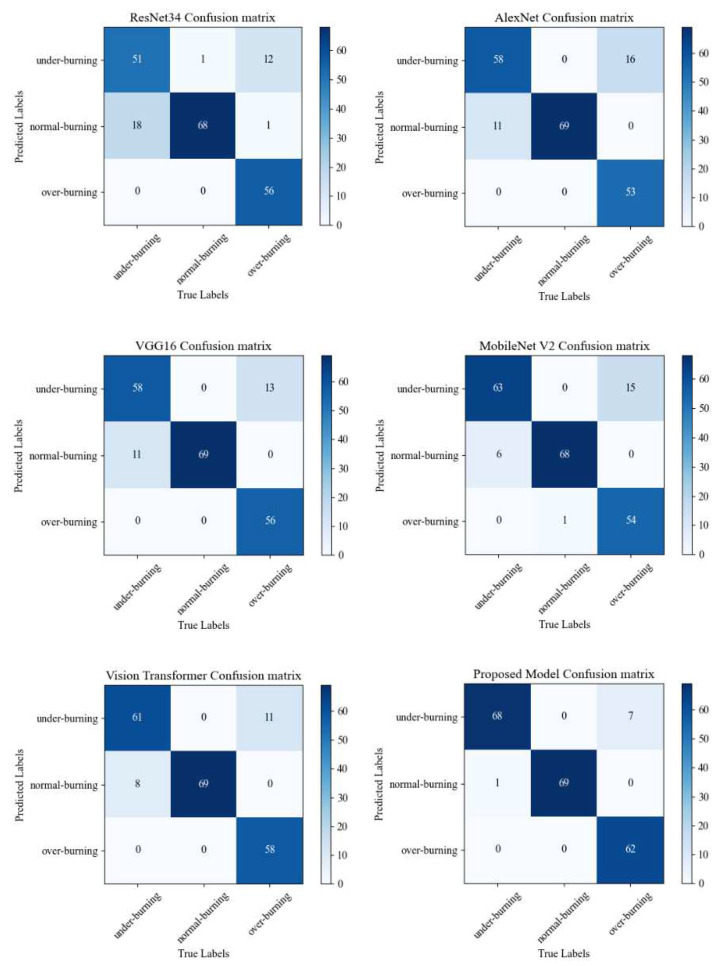
Heatmaps of different models.

In order to compare the performance of the above networks more comprehensively, a confusion matrix is adopted to reflect the actual recognition of each working condition category by different networks, as shown in Figure 11.

Figure 11 is the heatmaps of different models, which shows the total correct predictions of the models from total test images. 175 images out of 207, 180 images out of 207, 183 images out of 207, 185 images out of 207, 188 images out of 207 and 199 images out of 207 are correctly predicted by ResNet34 model, AlexNet model, VGG16 model, MobileNet V2 model, Vision Transformer model and the proposed model, respectively.

In Table 4, the details of the ResNet34 score are given. The score shows the precision, sensitivity and specificity.

Table 5 shows precision, sensitivity and specificity of AlexNet.

Table 6 shows precision, sensitivity and specificity of VGG16.

Table 7 shows precision, sensitivity, and specificity of MobileNet V2.

Table 8 shows precision, sensitivity and specificity of Vision Transformer.

Table 9 shows precision, sensitivity and specificity of Proposed Model.

**Figure 12:**
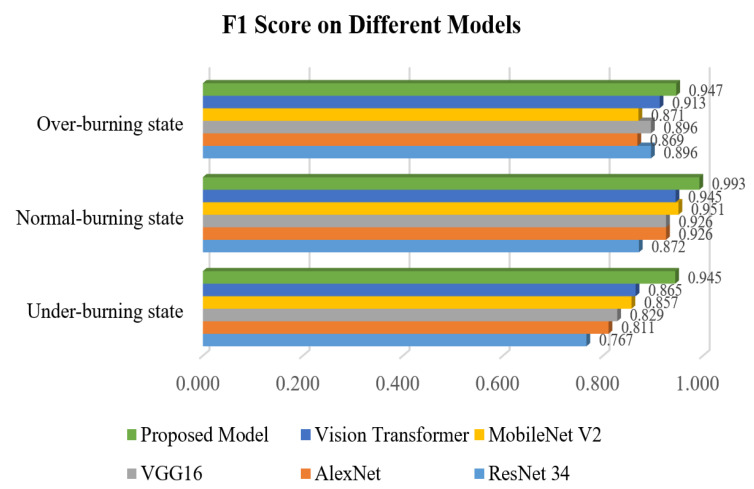
F1 score of working conditions on different models.

Figure 12 shows the visualization results of the F1-score in various working conditions on different models.

Table 10 shows the top-1 accuracy, number of parameters and training time of different models. Compared with other models, the proposed model has better accuracy and performance. The proposed model achieves 96.14% accuracy, and the cost of training time is also relatively small.

## 5. Discussion

The method proposed in this paper uses a deep learning model combined with transfer learning and CA attention module to help extract key features of flame images while reducing training costs. It can also be seen in Figure 6 and Table 3 that the addition of the attention module improves the condition recognition ability of the model, indicating that the addition of the attention module can effectively improve the ability of the network to extract key features of flame images. As can be seen from the number of parameter changes in the table, increasing the attention module will hardly increase the complexity of the model, and improve the performance of the model at a small cost. Combined with the visual analysis in Figure 7, it can be seen that the recognition model, after adding the CA module, expands the range of feature areas through network learning and gradually finds the important features that are conducive to distinguishing categories on the image. Then, the attention module is used to improve the network’s attention to important features and increase the corresponding feature weights.

According to the various evaluation indexes of different models in Figure 9, Figure 10, Figure 11 and Figure 12 and Table 4, Table 5, Table 6, Table 7, Table 8 and Table 9, such as precision, sensitivity, specificity, heatmaps, and F1 score, it can be seen that all networks have more misjudgments on the understanding of under-burning state and over-burning state. This is because the flame of these two conditions is similar, and it is difficult to classify them. However, the model proposed in this paper has a good ability to recognize the three types of working conditions. It can be seen from Table 10 that on the flame image dataset: ResNet34 achieves a top-1 accuracy of 84.54%; AlexNet reached 86.47%; The top-1 accuracy of VGG16 and MobileNetV2 in the test set reaches about 90%, but VGG16 has a large number of parameters and spends more time during training process; Vision Transformer reached 94.20%, but the model structure is relatively complex and the training takes up more resources, so it is difficult to converge.

The accuracy of the proposed model is 96.14%, and the recognition accuracy is slightly higher than that of the Vision Transformer. However, the complexity of the model is low, the running time takes up less resources and the training time cost is relatively small, indicating that the proposed model has a better performance in the task of condition recognition.

## 6. Conclusions

A deep learning network model combining transfer learning and coordinate attention mechanism for rotary kiln working condition recognition is proposed in this paper. Experiment results show that using transfer learning for deep learning network models can realize parameter sharing and feature transfer, speed up model convergence, reduce the dependence of model training on sample data and solve the problem of small sample learning. The introduction of the coordinate attention mechanism can improve the weight of key features and obtain accurate location information of the interest region. The improved model is helpful to improve the accuracy of the working condition recognition of rotary kilns. The limitations of the model are mainly the practical application of deployment on mobile devices. In future work, it is hoped that the model can be further improved by processing the flame image and reducing the size of the model. This will help with deploying the model on mobile devices.

## Figures and Tables

**Table 1:** Flame image samples.

Image Type	Number of Training Samples	Number of Test Samples
Under-burning state	279	69
Normal-burning state	281	69
Over-burning state	268	69

**Table 2:** Experimental parameters.

Experimental Parameters	Number of Training Samples
Learning rate	Freeze phase 1 × 10^−3^, Unfreeze phase 1 × 10^−4^
Batch size	Freeze phase 32, Unfreeze phase 16
Epochs	100

**Table 3:** Different attention module performance comparison.

Settings	Top-1 Acc (%)	Params (M)
ResNet50	92.75	25.6
+SE	93.24	28.1
+CBAM	93.72	28.1
+ECA	94.20	25.6
+CA	94.69	28.5

**Table 4:** ResNet34 Precision, Sensitivity and Specificity.

Working Class	Precision	Sensitivity	Specificity
Under-burning state	0.797	0.739	0.906
Normal-burning state	0.782	0.986	0.862
Over-burning state	1.0	0.812	1.0

**Table 5:** Shows precision, sensitivity and specificity of AlexNet.

Working Class	Precision	Sensitivity	Specificity
Under-burning state	0.784	0.841	0.884
Normal-burning state	0.863	1.0	0.920
Over-burning state	1.0	0.768	1.0

**Table 6:** Shows precision, sensitivity and specificity of VGG16.

Working Class	Precision	Sensitivity	Specificity
Under-burning state	0.817	0.841	0.906
Normal-burning state	0.863	1.0	0.920
Over-burning state	1.0	0.812	1.0

**Table 7:** MobileNet V2 Precision, Sensitivity and Specificity.

Working Class	Precision	Sensitivity	Specificity
Under-burning state	0.808	0.913	0.891
Normal-burning state	0.919	0.986	0.957
Over-burning state	0.982	0.783	0.993

**Table 8:** Vision Transformer Precision, Sensitivity and Specificity.

Working Class	Precision	Sensitivity	Specificity
Under-burning state	0.847	0.884	0.920
Normal-burning state	0.896	1.0	0.942
Over-burning state	1.0	0.841	1.0

**Table 9:** Proposed Model Precision, Sensitivity and Specificity.

Working Class	Precision	Sensitivity	Specificity
Under-burning state	0.907	0.986	0.950
Normal-burning state	0.986	1.0	0.993
Over-burning state	1.0	0.899	1.0

**Table 10:** Comparison of test results with different models.

Model	Top-1 Acc (%)	Params (M)	Training Time (s)
ResNet 34	84.54	11.7	1569.56
AlexNet	86.47	61.1	2283.09
VGG16	88.89	138.4	3521.74
MobileNet V2	90.34	3.5	1668.15
Vision Transformer	94.20	86	2097.81
Proposed Model	96.14	28.5	1863.24

## Data Availability

All data has been presented in main text.
